# Behavioral Health Risk Factors and Motivation to Change among Cardiovascular General Hospital Patients Aged 50 to 79 Years

**DOI:** 10.3390/nu14091963

**Published:** 2022-05-07

**Authors:** Ulrike Siewert-Markus, Sabina Ulbricht, Beate Gaertner, Birgit-Christiane Zyriax, Marcus Dörr, Stefanie Tobschall, Sophie Baumann, Ulrich John, Jennis Freyer-Adam

**Affiliations:** 1 Institute for Medical Psychology, University Medicine Greifswald, 17475 Greifswald, Germany; stefanie.tobschall@med.uni-greifswald.de (S.T.); jennis.freyer-adam@med.uni-greifswald.de (J.F.-A.); 2 German Centre for Cardiovascular Research (DZHK), Partner Site Greifswald, 17475 Greifswald, Germany; sabina.ulbricht@med.uni-greifswald.de (S.U.); marcus.doerr@med.uni-greifswald.de (M.D.); sophie.baumann@med.uni-greifswald.de (S.B.); ulrich.john@med.uni-greifswald.de (U.J.); 3 Department of Prevention Research and Social Medicine, Institute for Community Medicine, University Medicine Greifswald, 17475 Greifswald, Germany; 4 Robert-Koch-Institute, 13353 Berlin, Germany; gaertnerb@rki.de; 5 Preventive Medicine and Nutrition, Institute for Health Service Research in Dermatology and Nursing (IVDP), University Hospital Hamburg-Eppendorf, 20246 Hamburg, Germany; b.zyriax@uke.de; 6 German Centre for Cardiovascular Research (DZHK), Partner Site Hamburg, 20251 Hamburg, Germany; 7 Department of Internal Medicine B, University Medicine Greifswald, 17475 Greifswald, Germany; 8 Section Methods in Community Medicine, Institute for Community Medicine, University Medicine Greifswald, 17475 Greifswald, Germany

**Keywords:** older adults, dietary behavior, lifestyle, health behavior, cardiovascular disease, prevention

## Abstract

Little is known about the (co-)occurrence of smoking, alcohol at-risk drinking, physical inactivity and overweight, and the motivation to change these behavioral health risk factors (HRFs) in older general hospital patients with cardiovascular disease. Between October and December 2016, all consecutively admitted patients aged 50 to 79 years were proactively recruited on 3 cardiology wards and asked to participate in a survey on HRFs and behavior change motivation. Of the eligible patients, 80.4% participated in the survey (*n* = 328). The mean age was 66.5 years (standard deviation 9.0), and 65.5% were male. At least 1 HRF was present in 91.8% (*n* = 280), at least 2 HRFs in 54.4% (*n* = 166), and 3 or 4 HRFs in 12.1% (*n* = 37) of participants. The proportion of older adults who contemplated or were changing or planning to change their behavior to meet health behavior recommendations ranged between 66.0% (smoking) and 93.2% (alcohol consumption). The results indicate a notable co-occurrence of behavioral HRFs in older patients with cardiovascular disease. The majority of older adults were at least considering changing the respective behavior. To prevent and treat diseases efficiently, hospitalization may be a suitable moment for systematic multiple HRF screening and intervention.

## 1. Introduction

With the demographic aging of the population and an increasing life expectancy [1], maintaining health, physical and cognitive functioning, and independence in late life is an important public health goal [2]. In older age, most of the disease burden is due to non-communicable diseases, in particular cardiovascular disease. The 4 modifiable behavioral health risk factors (HRFs) of smoking, alcohol at-risk drinking, physical inactivity, and overweight are major contributors to the development of non-communicable diseases and to all-cause mortality [3]. A smaller number of HRFs are associated with lower morbidity rates [4,5,6], a lower mortality risk [7,8,9,10], in particular a lower cardiovascular disease incidence and mortality [7,10,11], better self-rated health [12], a slower rate of functional decline and better recovery from functional impairment and a delayed onset of disability [13,14,15,16,17], and a slower rate of cognitive decline and lower dementia risk [18,19,20]. Further, it has been found that nutritional status is related to the prognosis and length of hospital stay in patients with acute coronary syndrome or heart failure [21,22].

A general population study found that of the aforementioned 4 behavioral HRFs, 2 or more were present in 53.4% of European adults aged 50 years or over. The combination of overweight and physical inactivity had the highest prevalence of 35.4% [23]. A recent study, based on a large representative German general population sample, showed that the risk of having 2 or more, i.e., multiple, HRFs and of being overweight and/or physically inactive increased with age [24]. Only a few studies investigated multiple HRFs in older adults, and the findings are rather inconsistent concerning (a) which HRFs cluster or co-occur and (b) whether older adults tend to have a smaller or higher number of HRFs compared to younger adults [25,26]. 

It is expected that individual and co-occurring behavioral HRFs are more common among older general hospital patients with cardiovascular disease than in the general population of the same age as the HRFs may have contributed to hospital admission [27,28,29,30]. However, this representative data is lacking. 

To address behavioral HRFs, the general hospital has been found to be particularly suitable [31]. Hospitalization itself may be a health event that might motivate individuals to change unhealthy behaviors [32,33]. However, little is known about the motivation for behavioral changes among older adults in general and in particular among those with cardiovascular disease. A study in acute coronary syndrome patients showed that their behavioral HRFs greatly decreased 6 months after the beginning of inpatient cardiac rehabilitation while no further changes were detected after 1 year. Moreover, patients with multiple HRFs were less likely to maintain a healthier lifestyle over time [34]. 

This study had 3 aims: (1) to determine the occurrence and co-occurrence of 4 behavioral HRFs in older general hospital patients with cardiovascular disease; (2) to identify the association of sex, age, and school education with HRF number; and (3) to investigate the stages of change regarding recommended health behaviors in case of the presence of the respective HRF.

## 2. Materials and Methods

Data were obtained in a cross-sectional electronic survey on behavioral HRFs among general hospital patients with cardiovascular disease, which was approved by the Ethics Committee of the University Medicine Greifswald (BB 148/15, BB 067/16).

### Sampling Frame and Participants 

This study was conducted at the University Medicine Hospital Greifswald in northeastern Germany. During 10 weeks between 10 October and 16 December 2016, participants were recruited on 3 cardiology wards at the Department of Internal Medicine. On weekdays, all consecutively admitted patients in the predefined age range of 18 to 79 years were approached by 1 of 3 research assistants and asked to respond to survey questions regarding behavioral HRFs. Those who provided verbal informed consent for anonymous participation in the survey received an electronic handheld device and a brief introduction to the handling of the computerized self-administered questionnaire. The electronic handheld device also assessed electronic consent.

**Figure 1:**
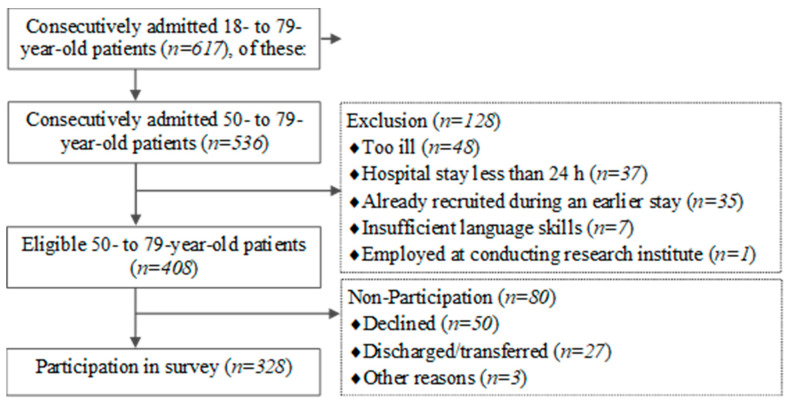
Participant flow chart.

Of 617 consecutively admitted patients, 536 were in the age range 50 to 79 years considered in the present analysis. Of these, those who were cognitively or physically incapable or terminally ill or had highly contagious diseases (*n* = 48), were discharged or transferred outside the study area within the first 24 h (*n* = 37), had already been recruited for a study during an earlier hospital stay (*n* = 35), had insufficient language skills (*n* = 7), and were employed at the conducting research institute (*n* = 1) were excluded from survey participation. This resulted in 408 patients who were eligible to participate in the survey, and 328 patients (80.4%) took part (Figure 1).

## 3. Measures

### 3.1. Behavioral Health Risk Factors

Four behavioral HRFs were assessed, namely current smoking, alcohol at-risk drinking, physical inactivity, and overweight. Each of the 4 HRFs was coded as 1 if it was present and 0 if it was absent, which resulted in a total HRF number of 0 to 4. 

Tobacco smoking (henceforth smoking) was assessed according to the question, “Do you currently smoke? Please answer the question with regard to the last 4 weeks before your hospital admission”. The response categories differentiated between daily smoking, occasional smoking, former smoking, and never smoking. Current occasional and daily smokers were considered smokers.

Alcohol at-risk drinking (henceforth alcohol) was determined using the total score of the Alcohol Use Disorder Identification Test-Consumption [35]. It consists of 3 items assessing the frequency and quantity of alcohol drinking and the frequency of heavy episodic alcohol drinking. According to Higgins-Biddle and Babor (2018), a gender-specific version for heavy episodic drinking was used: women/men were asked how often they had 4/5 or more alcoholic drinks on 1 occasion [36]. The total score ranged from 0 to 12. Alcohol at-risk drinking was considered present for women/men with a score of ≥4/5 [37]. These cut-offs correspond well with the national limits defined for healthy adults, i.e., >12/24 g of pure alcohol per day and >3/4 drinks per occasion for women/men [38,39]. There are no recommendations specific to older adults. Persons with acquired diseases, including cardiovascular disease, and persons taking medication should reduce their alcohol consumption [39,40].

Physical inactivity (henceforth inactivity) was assessed according to the International Physical Activity Questionnaire-Short Form [41], which assesses aerobic physical activity in the last 7 days. Due to the fact that patients were hospitalized when they were interviewed, the timeline was adapted in this study and referred to a typical week to account for illness-related physical activity restrictions before the hospital stay. In order to determine physical inactivity, the total amount of minutes per week of moderate and vigorous physical activity was calculated. Those with ≥150 min per week of moderate activity, ≥75 min per week of vigorous activity, or an equivalent amount of both were subsumed to be sufficiently physically active in accordance with the recommendations of the World Health Organization (WHO) for both adults and older adults [42], which largely agree with recommendations specifically tailored to older adults [43,44]. Those who did not reach this level of activity were referred to as physically inactive.

Overweight or obesity (henceforth overweight) was assessed based on the body mass index (BMI) obtained from participants’ self-reported weight and height. BMI is defined as body weight in kilograms, divided by body height in square meters. BMI ≥ 25.0 was defined as overweight or obesity [45]. This definition is based on the WHO recommendations for adults due to the lack of specific recommendations for older adults.

### 3.2. Motivation to Change Behavioral Health Risk Factors

If a certain behavioral HRF was present, the stage of change according to the Transtheoretical Model of Intentional Behavior Change (TTM) was assessed for smoking cessation, alcohol low-risk drinking, sufficient physical activity, and a healthy diet, respectively. According to TTM, persons proceed through stages of change; from not thinking about change (pre-contemplation), through being ambivalent about change (contemplation), planning to change (preparation), and manifesting change (action) to maintaining achieved change (maintenance) [46]. For each relevant health behavior, an adapted 4-item measure was used [47,48]. It allocates individuals to (1) action, i.e., participants who reported a lasting attempt to adhere to the respective recommendation within the past 6 months. Thus, the action stage included participants who, despite their efforts to reduce or increase the respective behavior, still did not meet the recommendations. The action stage was not assessed among smokers as persons in action would have reported current non-smoking, and the stage of change was not assessed in current non-smokers. All others were allocated to: (2) pre-contemplation, i.e., participants who did not intend to permanently adhere to the respective health behavior recommendation; (3) contemplation, i.e., participants who considered permanently adhering to the recommendation; and (4) preparation, i.e., participants who seriously planned to permanently adhere to the recommendation.

## 4. Other Measures

Since BMI is a simplified measure and indicator of an unhealthy diet, compliance with the recommendations of a Mediterranean diet, namely high consumption of vegetables, fruits, fish, nuts, legumes, and olive or rapeseed oil, and low consumption of meat, meat products, sweets, and sweetened beverages, was assessed with questions adapted from the Mediterranean Diet Adherence Screener [49], as shown in Supplementary Table S1.

Self-rated health was assessed using the single item “How would you describe your health in general?”, and the response categories (1) “excellent”, (2) “very good”, (3) “good”, (4) “fair”, and (5) “poor”. For the analysis, the responses were dichotomized in lower self-rated health (4 to 5) versus better self-rated health (1 to 3).

Sociodemographic characteristics included self-reported sex, age in years, and highest school education. Age was collapsed into the 3 age groups of 50 to 59, 60 to 69, and 70 to 79 years. Two levels of school education achievement were distinguished: a lower level (school leaving certificate after 10th grade or earlier) and higher level (school leaving certificate after 11th grade or later).

### Statistical Analysis

Proportions and 95% confidence intervals (CIs) are given. Proportions for the 4 single behavioral HRFs and for 1 or more HRF, 2 or more (multiple) HRFs, and 3 or 4 HRFs are presented in total and stratified by sociodemographic characteristics. Proportions of 0 HRFs and 15 HRF patterns, i.e., specific combinations of HRFs, are presented for the total sample. To investigate variables associated with HRF number (range 0 to 4), a multivariable ordered logistic regression analysis with sex, age in years, and school education as predictors was calculated. Furthermore, the proportions and CIs of the 4 stages of change for each health behavior are presented. Finally, additional analyses tested the compliance with the recommendations of a Mediterranean diet of non-overweight and overweight/obese participants. *p*-values < 0.05 and non-overlapping CIs were considered statistically significant. STATA version 14.2 SE was used. Cases with missing values were excluded list-wise.

## 5. Results

### 5.1. Sample Characteristics

Overall, participants were, on average, 66.5 years old (*n* = 328; standard deviation, SD = 9.0, median = 67.0), 65.5% were men, 70.4% had a lower level of school education, and 61.9% reported better self-rated health. In the total sample, the mean HRF number was 1.6 (SD = 0.8, median = 2.0; Table 1).

### 5.2. Occurrence and Co-Occurrence of Behavioral Health Risk Factors

Overweight was the most common HRF (75.9%, *n* = 249), followed by inactivity (49.5%, *n* = 153), alcohol (19.0%, *n* = 59), and smoking (16.1%, *n* = 50; Table 2). The HRF alcohol was more common in men (24.0%, 95% CI 18.6 to 30.4, *n* = 49) than in women (9.3%, 95% CI 5.0 to 16.7, *n* = 10), and in 50- to 59-year-old participants (31.0%, 95% CI 22.1 to 41.7, *n* = 27) compared to those aged 70 to 79 years (12.9%, 95% CI 8.1 to 19.8, *n* = 17). Smoking was more common in 50- to 59-year-old (29.9%, 95% CI 21.1 to 40.5, *n* = 26) and 60- to 69-year-old (19.6%, 95% CI 12.6 to 29.1, *n* = 18) participants compared to those aged 70 to 79 years (4.5%, 95% CI 2.0 to 9.8, *n* = 6).

In the total sample, 8.2% of the participants had no behavioral HRF (*n* = 25), 37.4% had 1 HRF (*n* = 114), 42.3% had 2 HRFs (*n* = 129), and 11.5% (*n* = 35) had 3 HRFs (Table 3). Among the 5 most common HRF patterns, 4 contained overweight: overweight alone (25.9%, *n* = 79), overweight plus inactivity (29.2%, *n* = 89), overweight plus alcohol (7.5%, *n* = 23), overweight plus inactivity plus smoking (4.6%, *n* = 14), and inactivity alone (8.5%, *n* = 26).

### 5.3. Associations of Sociodemographic Characteristics and Number of HRFs

The proportions of at least 1 HRF, 2 or more HRFs, and 3 or more HRFs by socio-demographics are shown in Table 4. The multivariable ordered logistic regression analysis revealed that the number of HRFs was inversely associated with age (OR 0.97, 95% CI 0.95 to 0.996, *p* = 0.02) and school education (OR 0.60, 95% CI 0.37 to 0.97, *p* = 0.04, reference: lower school education).

### 5.4. Motivation to Change Regarding Recommended Health Behaviors

As depicted in more detail in Table 5, 81.6% (*n* = 199) of the participants who were overweight contemplated or planned or were attempting to follow a healthier diet. Of the participants who were inactive, 84.9% (*n* = 129) and 93.2% (*n* = 55) of the participants with at-risk alcohol consumption contemplated or planned or were attempting to change their behavior to meet respective recommendations. Of the participants who were current smokers, 66.0% (*n* = 33) were at least considering or planning to quit smoking.

Considering the most common combination of 2 HRFs, i.e., overweight and inactivity, 49.4% (95% CI 39.0 to 59.9; *n* = 44) of the participants were in the same stage of change for the respective health behavior recommendations, 18.0% (95% CI 11.2 to 27.6; *n* = 16) were in a higher stage for healthy diet recommendations, and 32.6% (95% CI 23.5 to 43.2; *n* = 29) were in a higher stage for physical activity recommendations.

### 5.5. Additional Analyses of Dietary Habits

Supplementary Table S1 presents a more detailed description of the dietary habits, beyond the BMI categories, which served as a simplified indicator of an unhealthy diet. Information is provided regarding the food categories of the Mediterranean diet for the total sample and separately for non-overweight and overweight participants. In the total sample, non-adherence to different food recommendations ranged between 40.9% (limiting intake of sweets) and 99.4% (sufficient intake of olive or rapeseed oil). Independent of weight status, adherence to the Mediterranean diet recommendations was low. 

## 6. Discussion

Three main findings indicate a high need for multiple HRF screening and intervention in general hospital patients aged 50 to 79 years with cardiovascular disease. Firstly, 54% of all patients reported multiple behavioral HRFs, with younger and less educated patients reporting a higher number of HRFs. Secondly, among the 5 most common HRF patterns, 4 contained overweight and 3 inactivity, as single HRFs or in combination with other HRFs. Thirdly, 66% and more of the participants with the respective HRFs were at least considering, planning, or attempting to change the respective HRF. 

The findings concerning the high co-occurrence of multiple HRFs among 50- to 79-year-old general hospital patients with cardiovascular disease are in line with findings in the 50-year-old or older general population showing that 2 or more HRFs were present in more than 50% of the population [23,24]. Further, around 90% of both our cardiologic patients and the general population in this age range had at least 1 HRF [23,24]. These findings were confirmed by a recent study in the general German population aged 45 years and over, in which over 90% of the participants confirmed at least 1 of 5 HRFs [50]. 

Moreover, in line with general population studies, we found a higher number of HRFs in less educated participants compared to higher educated participants [24,25,26,51]. In addition, younger age was associated with a higher number of HRFs. This contradicts general adult population studies, which found a higher risk of having 1 or more HRFs with increasing age [24,50]. However, the findings concerning age are rather inconsistent [25,26]. We did not find a higher number of HRFs in men compared to women as shown in previous general population studies [24,25,26,51]. However, due to our comparatively small sample size, this result should be interpreted with caution. Discrepancies in how many and which HRFs were considered and how they were assessed and defined hinder a comparison of results, and different study populations were also used [24,25]. 

Regarding single health behaviors, the occurrence of behavioral HRFs in older general hospital patients with cardiovascular disease was similar to the HRF occurrence in the general population [51]. The present study showed that at 75%, obesity was by far the most common HRF, followed by physical inactivity at 50%. In comparison, a study on European adults aged 50 years or older showed a lower occurrence of overweight (60%) and a higher occurrence of physical inactivity (71%). With regard to smoking, the occurrence was comparable between the 2 studies (16% versus 18%). With regard to risky alcohol consumption, the occurrence in the present study was significantly higher (19% versus 4% [51]).

In line with previous findings, the most common pattern of HRFs in older patients was overweight and inactivity (29% versus 35% in [23]). Findings in patients with coronary heart disease also revealed a high co-occurrence of overweight and inactivity [52]. While overweight (26% versus 13%) and inactivity (9% versus 22% in [23]) as single HRFs accounted for the second and third most common HRF patterns in our study, overweight plus alcohol at-risk drinking (8% versus 1% [23]) and overweight plus inactivity plus smoking (circa 5% in both studies) were the fourth and fifth most common HRF patterns, respectively. Reasons for reduced physical activity and increased overweight in older age groups could include bodily complaints, lack of training, changes in body composition, and a more sedentary lifestyle compared to younger people. Reduced smoking and alcohol at-risk drinking could be due to quitting smoking and alcohol consumption when facing ill health and also due to selective mortality as a result of smoking and alcohol consumption and related health disturbances [24]. Overall, the HRFs overweight and alcohol at-risk drinking were more common in the patient sample. 

Our study provides a first insight into the motivation underlying behavior change in older individuals with cardiovascular disease. With 66% of those who smoked and more than 80% of those who were alcohol at-risk drinkers, inactive, or overweight contemplating or planning to change, or changing their behavior to meet respective recommendations, motivation to change was high. A study of recently discharged 17- to 96-year-old general hospital patients reported a higher proportion of participants who were at least considering to change their behavior for smoking (80%), lower respective proportions for alcohol at-risk drinking (52%) and physical inactivity (61%), and a comparable proportion for overweight (86%) [53]. These differences could be due to a wider age range and a lower participation rate (59% versus 79% in our study), which may have resulted in the recruitment of particularly motivated patients in Haynes (2008). With regards to the higher motivation to change at-risk alcohol use and to adhere to physical activity recommendations, our algorithm may have overestimated the proportion of participants in the action stage, as the participants were first assigned to action, and only those participants who did not report a lasting attempt regarding behavior change were allocated to the earlier motivational stages. As these participants still had the corresponding HRF, they may not have been aware of this.

Showing a high (co-)occurrence of behavioral HRFs among older general hospital patients with cardiovascular disease and a low fulfillment of nutritional recommendations, our findings support previous findings suggesting that diagnosis and routine care alone may not be sufficient in helping patients change their lifestyle accordingly. Dietary changes are especially challenging because dietary recommendations are generally complex, hard to recall, and could lead to confusion and suboptimal diet change [54]. Educating patients on diet prophylaxis is of great importance as nutritional status is related to the prognosis and length of hospitalization of cardiovascular disease patients [21,22]. Professional counseling is especially important to prevent muscle mass loss and frailty [55]. A multiple-HRF intervention approach may be central to reverse or delay declines in physical and cognitive functioning in older adults [2]. Tailored to each person’s current stage of change, a multiple-HRF intervention might lead to more effective health promotion than interventions focusing on single-behavior change [56,57]. A study on community-dwelling individuals aged 65 years or older in primary care showed that health risk assessment combined with computer-generated feedback reports and counselling was promising in terms of improved health behaviors and prolonged survival [58]. Within a randomized controlled trial, a multiple-HRF intervention approach improved or maintained cognitive functioning in at-risk people aged 60 to 77 years from the general population [59]. Within the multiple-HRF intervention, dietary improvement due to dietary counselling was associated with beneficial changes in executive function [60].

## 7. Strengths

The strengths of this study include, firstly, the high proportion of eligible patients who participated in the study (79%), thus minimizing the risk of selection bias. Secondly, this study investigated not only the occurrence of single HRFs but also the co-occurrence of 4 behavioral HRFs known to be major contributors to the development and maintenance of non-communicable diseases, such as cardiovascular disease, in patients. Thirdly, this study reveals first insights into the stages of behavioral change motivation of older general hospital patients with regard to recommended health behaviors. 

## 8. Limitations

Several limitations of this study need to be considered. Firstly, the sample size was small for testing subgroup differences and associations. Distinct differences in the specific HRF patterns were shown in previous general population studies, between women and men, age groups, and different levels of education [24,25], which could only be partly analyzed in the present study. However, further consideration of these differences in older populations and specifically in older general hospital patients should be the subject of future research. Secondly, the occurrence of HRFs may be underestimated as the health behavior recommendations may not apply in detail to older individuals. For example, we might have underestimated the HRF alcohol at-risk drinking in our sample because the national recommendation for alcohol consumption only applies to healthy individuals [39]. Certain diseases or medication intake, which are found more often in older individuals, might contradict alcohol consumption at all. In addition, due to age changes in body composition, including higher fat mass and lower muscle mass proportions, alcohol is metabolized slower and alcohol consumption should generally be reduced by older individuals. We may also have underestimated physical inactivity due to socially desirable self-reported answers. Over-reporting of physical activity is a common problem, even with validated instruments [61]. Further, self-reported statements, as used in our study, are likely to underestimate overweight [62]. Nevertheless, for behavioral HRFs, self-report remains the most widespread and feasible method of assessment [63]. Thirdly, in our study, both overweight and non-overweight participants reported a high non-adherence to the recommendations of the Mediterranean diet [49], showing that overweight as a proxy variable for an unhealthy diet might be an oversimplified and insufficient measurement. Fourthly, the generalizability of our findings to patients from other departments or to internal medicine departments of other general hospitals in Germany or other countries may be limited. Fifthly, this study did not consider the patients’ medical conditions, which could be related to whether patients adhere to a healthy lifestyle and the stage of behavioral change motivation they are in. In addition, although HRF occurrence was studied interdependently, how the stages of change coincided or diverged when multiple HRFs co-occurred was not determined.

## 9. Conclusions

With multiple behavioral HRFs being present in 54% of the patients, our study demonstrated a high need for systematic multiple-HRF screening and intervention in older hospital patients with cardiovascular disease. The overall occurrence and co-occurrence of behavioral HRFs was high, particularly in younger and less educated patients. Despite the high occurrence of HRFs found, their occurrence may still be underestimated as it was determined based on the health behavior recommendations for healthy people. 

## 10. Clinical Implications

Although the HRFs smoking and alcohol at-risk drinking were found to be less frequent in older age groups, they were still not negligible. The energy-balance-related HRFs overweight, inactivity, and nutrition should be the special focus of treatment and preventive efforts. Particularly, low compliance with healthy diet recommendations suggests a high demand for nutritional advice. The implementation of systematic proactive multiple-HRF screening and intervention in routine hospital care could reach all patients who are, despite their age, largely motivated to change behavioral HRFs. It may serve secondary prevention purposes by improving treatment success in patients as, at this point, health care still lacks systematic preventive measures that are accessible to all patients. Consequently, significant potential to improve individual prognosis is still being missed.

List of abbreviations: CI: confidence interval; HRF: health risk factor; M: mean: SD: standard deviation.

## Figures and Tables

**Table 1:** Sociodemographic characteristics, self-rated health, and number of behavioral health risk factors (of the four health risk factors of overweight, inactivity, alcohol, and smoking) for the total sample and stratified by sex.

	Total	Men	Women
***n* (%)**	328	215 (65.5)	113 (34.5)
**Age in years**			
M (SD)	66.5 (9.0)	65.9 (9.2)	67.7 (8.4)
Median (IQR)	67.0 (59.0–75.0)	66.0 (58.0–75.0)	69.0 (61.0–75.0)
School education level *			
Lower, *n* (%)	214 (70.4)	144 (72.0)	70 (67.3)
Higher, *n* (%)	90 (29.6)	56 (28.0)	34 (32.7)
**Self-rated health**			
Better, *n* (%)	203 (61.9)	138 (64.2)	65 (57.5)
Lower, *n* (%)	125 (38.1)	77 (35.8)	48 (42.5)
Number of health risk factors **			
M (SD)	1.6 (0.8)	1.7 (0.9)	1.5 (0.7)
Median (IQR)	2.0 (1.0–2.0)	2.0 (1.0–2.0)	1.0 (1.0–2.0)

Notes: *n* = number, M = mean, SD = standard deviation, IQR = interquartile range. * *n* = 304; ** *n* = 305.

**Table 2:** Individual behavioral health risk factors (regardless of whether any other health risk factors were present) in the total sample and stratified by sociodemographic characteristics (percent, 95% confidence interval).

	Overweight		Inactivity		Alcohol		Smoking	
	*n*		*n*		*n*		*n*	
**Total**	328	75.9 (71.0–80.3)	309	49.5 (43.9–55.1)	311	19.0 (15.0–23.7)	311	16.1 (12.4–20.6)
**Sex**								
Men	215	76.7 (70.6–81.9)	202	48.0 (41.2–55.0)	204	24.0 (18.6–30.4)	204	18.6 (13.8–24.6)
Women	113	74.3 (65.4–81.6)	107	52.3 (42.8–61.8)	107	9.3 (5.0–16.7)	107	11.2 (6.4–18.9)
**Age (years)**								
50–59	92	73.9 (63.8–82.0)	85	43.5 (33.2–54.4)	87	31.0 (22.1–41.7)	87	29.9 (21.1–40.5)
60–69	94	84.0 (75.0–90.2)	91	52.7 (42.3–62.9)	92	16.3 (10.0–25.5)	92	19.6 (12.6–29.1)
70–79	142	71.8 (63.8–78.7)	133	51.1 (42.6–59.6)	132	12.9 (8.1–19.8)	132	4.5 (2.0–9.8)
School education level *								
Lower	214	80.8 (75.0–85.6)	209	54.5 (47.7–61.2)	214	15.4 (11.1–20.9)	214	19.2 (14.4–25.0)
Higher	90	66.7 (56.1–75.8)	89	37.1 (27.5–47.7)	90	27.8 (19.4–38.1)	90	10.0 (5.2–18.3)

Notes: *n* = number; * Data available for *n* = 304 or *n* = 298 (inactivity) patients.

**Table 3:** Patterns of behavioral health risk factors in the total sample (*n* = 305).

Pattern of Health Risk Factor(s)	%	95% CI
0 health risk factors	8.2	5.6–11.9
Overweight	25.9	21.3–31.1
Inactivity	8.5	5.9–12.3
Alcohol	1.0	0.3–3.0
Smoking	2.0	0.9–4.3
1 health risk factor	37.4	32.1–43.0
Overweight plus inactivity	29.2	24.3–34.6
Overweight plus alcohol	7.5	5.1–11.1
Overweight plus smoking	2.6	1.3–5.2
Inactivity plus alcohol	1.0	0.3–3.0
Inactivity plus smoking	1.0	0.3–3.0
Alcohol plus smoking	1.0	0.3–3.0
2 health risk factors	42.3	36.8–47.9
Overweight plus inactivity plus alcohol	3.6	2.0–6.4
Overweight plus inactivity plus smoking	4.	2.7–7.6
Overweight plus alcohol plus smoking	2.3	1.1–4.8
Inactivity plus alcohol plus smoking	1.0	0.3–3.0
3 health risk factors	11.5	8.3–15.6
Overweight plus inactivity plus alcohol plus smoking	0.7	0.2–2.6

Notes: *n* = number, CI = confidence interval.

**Table 4:** Number of behavioral health risk factors in the total sample and stratified by sociodemographic characteristics (percent, 95% confidence interval).

		Behavioral Health Risk Factor(s)		
	*n*	≥1	≥2	≥3
**Total**	305	91.8 (88.1–94.4)	54.4 (48.8–60.0)	12.1 (8.9–16.3)
**Sex**				
Men	199	91.0 (86.1–94.2)	58.3 (51.3–65.0)	15.1 (10.7–20.8)
Women	106	93.4 (86.6–96.9)	47.2 (37.7–56.8)	6.6 (3.1–13.4)
**Age (years)**				
50–59	82	93.9 (85.9–97.5)	57.3 (46.2–67.7)	20.7 (13.2–31.1)
60–69	91	91.2 (83.2–95.6)	63.7 (53.2–73.1)	15.4 (9.2–24.5)
70–79	132	90.9 (84.6–94.8)	46.2 (37.8–54.9)	4.5 (2.0–9.8)
**School education level**				
Lower	209	95.7 (91.9–97.8)	58.4 (51.5–64.9)	12.0 (8.2–17.2)
Higher	89	83.1 (73.7–89.7)	44.9 (34.8–55.6)	13.5 (7.7–22.5)

Notes: *n* = number.

**Table 5:** Stages of change regarding recommended health behaviors for participants with the respective behavioral health risk factor (percent, 95% confidence interval).

Health Risk		Recommended Behavior	Stage of Change			
Factor Present	*n*		Pre-Contemplation	Contemplation	Preparation	Action
**Overweight**	244	Healthy diet	18.4 (14.0–23.9)	54.5 (48.2–60.7)	4.5 (2.5–8.0)	22.5 (17.7–28.3)
**Inactivity**	152	Sufficient physical activity	15.1 (10.2–21.8)	46.7 (38.8–54.7)	17.1 (11.9–24.0)	21.1 (15.2–28.3)
**Alcohol**	59	No or low-risk consumption	6.8 (2.5–17.1)	37.3 (25.7–50.6)	6.8 (2.5–17.1)	49.2 (36.4–62.1)
**Smoking**	50	Smoking cessation	34.0 (21.9–48.6)	40.0 (27.1–54.5)	26.0 (15.4–40.3)	/

Notes: *n* = number.

## Data Availability

On reasonable request, the data that support the findings of this study can be viewed and evaluated with the responsible researchers on site. To comply with the statement given in the informed consent procedure, the data cannot be passed on to third parties or uploaded for public access.

## Supplementary Materials

The following supporting information can be downloaded at: https://www.mdpi.com/article/10.3390/nu14091963/s1, Table S1: Average consumption of food components and non-adherence to dietary recommendations according to the Mediterranean Diet Adherence Screener (Martinez-Gonzalez, Garcia-Arellano et al. 2012) in the total sample, and stratified by non-overweight (BMI < 25) and overweight (BMI ≥ 25). Reference [49] is cited in the supplementary materials.
