# Supplementary material for: Behavioral Health Risk Factors and Motivation to Change among Cardiovascular General Hospital Patients Aged 50 to 79 Years
Source: Nutrients. 2022 May 7;14(9):1963. doi: 10.3390/nu14091963 (PMC9105822; doi:10.3390/nu14091963)
Supplement: Supplementary file 1 [file nutrients-14-01963-s001.zip › nutrients-1698019-supplement.pdf]

Table S1. Average consumption of food components and non-adherence to dietary recommendations according to the Mediterranean Diet Adherence Screener (Martinez-Gonzalez, Garcia-Arellano et al. 2012) in the total sample, and stratified by non-overweight (BMI<25) and overweight (BMI≥25).

Items	Descriptives	Total n=325	Non-overweight n=78	Overweight n=247
Do you mainly use olive or rapeseed oil for cooking?	% No (95% CI)	30.8 (26.0-36.0)	38.5 (28.2-49.9)	28.3 (23.0-34.3)
How many tablespoons of olive oil or rapeseed oil do you use on average per week?[1]	Mean (SD)	4.3 (5.3)	3.8 (4.6)	4.4 (5.5)
	Median (IQR)	3 (1-6)	2 (1-4)	3 (1-6)
	% <28 tbsp (95% CI)	99.4 (97.5-99.8)	100.0 (/)	99.2 (96.8-99.8)
How many vegetable servings do you usually consume per day? Please do not count potatoes! (1 serving: 200 g)[1] Examples of 1 serving: 2 medium sized carrots, 2-3 large tomatoes, ⅓-½ cucumber, 1 kohlrabi, 1 medium zucchini, 2 side dishes, 1 salad plate, 8-10 olives, 1 glass (0.2 l) or more vegetable juice	Mean (SD)	1.5 (1.1)	1.5 (1.6)	1.5 (0.9)
	Median (IQR)	1 (1-2)	1 (1-2)	1 (1-2)
	% <2 servings (95% CI)	62.1.(56.7-67.3)	71.4 (60.1-80.6)	59.2 (52.9-65.2)
How many fruit servings do you usually consume per day (1 serving: 80g)?[1] Examples of 1 serving: 1 small apple, 1 small banana, 1 small peach, ½ hand full of soft fruit, 1 small bowl of fruit salad, 1 small glass (0.1 l) or more juice with 100% fruit content. Please do not count nectar and fruit juice drinks!	Mean (SD)	2.0 (1.7)	2.3 (2.5)	1.9 (1.4)
	Median (IQR)	2 (1-3)	2 (1-3)	2 (1-3)
	% <3 servings (95% CI)	73.0 (67.8-77.6)	71.4 (60.1-80.6)	73.5 (67.5-78.7)
How many servings of meat (1 serving: 100-150 g) or meat products (25-30 g) do you consume per week?[2] Examples of 1 serving: 1 schnitzel, 1 roulade, 1 small steak or chop, 1 meatball, 1 bratwurst or 1-2 slices of mortadella, liver sausage, black pudding, ham, salami	Mean (SD)	9.5 (6.7)	9.5 (7.0)	9.5 (6.6)
	Median (IQR)	7 (7-14)	7 (7-14)	7 (7-14)
	% ≥7 servings (95% CI)	83.0 (78.4-86.7)	81.8 (71.3-89.1)	83.3 (78.1-87.5)
How many servings of butter, margarine, or cream do you consume per day (1 serving: 1 level tablespoon)?[2]	Mean (SD)	1.7 (1.5)	1.8 (1.1)	1.7 (1.5)
	Median (IQR)	1 (1-2)	1 (1-2)	1 (1-2)
	% ≥1 serving (95% CI)	90.7 (87.0-93.4)	96.1 (88.3-98.8)	89.0 (84.4-92.4)
How many sweet or carbonated beverages do you drink per day (1 beverage: 0,33 l)?[2] Please include fruit juice drinks and nectar here.	Mean (SD)	1.0 (1.4)	1.0 (1.3)	1.0 (1.4)
	Median (IQR)	0 (0-2)	1 (0-2)	0 (0-2)
	% ≥1 serving (95% CI)	47.4 (41.9-52.9)	50.6 (39.4-61.8)	46.3 (40.2-52.6)
How many servings of legumes (such as beans, peas, lentils) do you consume per week?[2] Examples of 1 serving: 1 small dish or 1 large trowel cooked legumes	Mean (SD)	1.1 (1.1)	1.0 (0.8)	1.1 (1.2)
	Med (IQR)	1 (0-2)	1 (1-1)	1 (0-2)
	% <3 servings (95% CI)	94.7 (91.7-96.7)	96.1 (88.3-98.8)	94.3 (90.6-96.6)
How many servings of fish (1 serving: 100-150 g) or seafood (1 serving: 200 g) do you consume per week?[2] Examples of 1 serving: 1 small salmon fillet, ½ mackerel, ½ herring, ½ gourmet filet, 1 large redfish fillet, 1 plaice, 1 trout, 1 sole, 1 fish bowl, 4-5 king prawns, 1 small box of mussels	Mean (SD)	1.5 (1.3)	1.3 (1.1)	1.5 (1.3)
	Median (IQR)	1 (1-2)	1 (1-2)	1 (1-2)
	% <3 servings (95% CI)	86.4 (82.2-89.7)	90.9 (81.9-95.7)	85.0 (79.9-88.9)

How many servings of sweets (1 serving: 20 g) or pastries (1 serving: 50-70 g) do you consume per week?[2]	Mean (SD)	3.0 (3.4)	3.2 (3.5)	2.9 (3.4)
	Median (IQR)	2 (1-4)	2 (1-4)	2 (1-3)
Examples of 1 serving: 4 candies, 20 small gummy bears, 1 small bar, 4 pieces of chocolate, 3-7 biscuits, 1 small croissant, 1 small piece of cake, ½ piece of tart	% ≥3 servings (95% CI)	40.9 (35.6-46.3)	42.9 (32.1-54.3)	40.2 (34.3-46.5)
How many servings of nuts (such as walnuts, hazelnuts, peanuts) do you consume per week (1 serving: 30 g)?[2]	Mean (SD)	1.1 (1.5)	1.3 (1.7)	1.1 (1.5)
	Median (IQR)	1 (0-2)	1 (0-2)	1 (0-2)
Examples of 1 serving: 1 small handful, 6-7 walnuts, 20 hazelnuts, 2-3 tablespoons chopped nuts	% <3 servings (95% CI)	86.1 (81.8-89.5)	83.1 (72.8-90.1)	87.0 (82.1-90.7)
Do you season 2 times a week or more often pasta, vegetable or rice dishes with garlic, tomatoes, leeks or onions?[2]	% No (95% CI)	23.5 (19.2-28.5)	24.7 (16.2-35.8)	23.2 (18.3-28.9)

Notes: n=Number, 95% CI=95% Confidence interval, SD=Standard deviation, IQR=Interquartil range, tbsp=tablespoon, g=gram, l=liter. [1] data available for n=322 patients, [2] data available for n=323 patients.

Martinez-Gonzalez, M. A., et al. (2012). "A 14-item Mediterranean diet assessment tool and obesity indexes among high-risk subjects: the PREDIMED trial." PLoS One **7**(8): e43134.
